# A Comprehensive Review of the Surface and Chromatic Properties of Monolithic Zirconia: Evaluating the Impact of Polishing and Finishing Methods on Aesthetics and Performance

**DOI:** 10.7759/cureus.66029

**Published:** 2024-08-02

**Authors:** Maaz Vohra, Kiran Kumar Pandurangan, Amrutha Shenoy, Varun Keskar

**Affiliations:** 1 Department of Prosthodontics, Saveetha Dental College and Hospitals, Saveetha Institute of Medical and Technical Sciences, Saveetha University, Chennai, IND

**Keywords:** color properties, surface properties, finishing and polishing, glazing, monolithic zirconia, quality of life, health

## Abstract

Monolithic zirconia is widely used in dentistry due to its outstanding mechanical properties, biocompatibility, and aesthetic qualities. This review examines how different polishing and finishing methods impact the performance and appearance of monolithic zirconia restorations. Derived from zirconium, zirconia is a robust ceramic that exists in monoclinic, tetragonal, and cubic forms, with properties that prevent crack propagation. Monolithic zirconia, preferred over porcelain-fused-to-metal (PFM) crowns, offers better aesthetics and avoids chipping. Various surface treatments, such as polishing and glazing, enhance zirconia’s smoothness and wear characteristics. Polished zirconia is less abrasive to enamel than glazed zirconia, making it more suitable for opposing teeth. Research indicates that polished zirconia has a smoother surface and higher fracture resistance compared to other dental ceramics. Surface roughness, which is influenced by the treatment method, is crucial in minimizing wear on opposing teeth. Polished monolithic zirconia also shows high flexural strength, chipping resistance, and translucency. While both polishing and glazing reduce brightness, polishing better preserves translucency. The literature identifies polishing as the best post-processing method for enhancing zirconia’s surface quality and mechanical properties without compromising its load-bearing capacity. In conclusion, polishing and finishing significantly improve the aesthetic and clinical performance of monolithic zirconia, confirming its effectiveness for durable and visually appealing dental restorations.

## Introduction and background

The name "zirconium" comes from the Arabic word "Zargun," meaning "golden in color," which is derived from Persian words for gold ("zar") and color ("gun") [1]. Zirconium is a transition metal with atomic number 40. When purified, it is a silver-colored metal resistant to corrosion [2]. When combined with oxygen, it forms zirconia, a strong and biocompatible ceramic. Zirconia is a highly aesthetic material used in dentistry due to its superior transformation toughening properties, the best among all dental ceramics. It also offers a more natural appearance compared to metal ceramics [3]. Zirconia is often used in making prosthetic devices due to its excellent chemical properties, high mechanical strength, stability, toughness, and Young's modulus of 210 GPa, which is close to the 193 GPa of stainless steel [4].

Zirconia can exist in three crystal forms: monoclinic, tetragonal, and cubic. At room temperature, it is in the monoclinic form, changing to tetragonal at 1170°C, and then to cubic at 2370°C. When zirconia transitions from the tetragonal to the monoclinic phase, it undergoes a notable volume increase of 3%-5% [5]. This expansion generates compressive stresses that help inhibit and reduce the spread of cracks. Under in vitro conditions, zirconia exhibits a flexural strength ranging from 900 to 1200 MPa and a fracture toughness of approximately 9 to 10 MPa/m² [6]. As a biocompatible, insoluble metal oxide with favorable radiopacity, and low corrosion potential, zirconia, when partially stabilized with yttrium, lacks a glass phase and instead has a highly crystalline structure with low translucency [7].

In recent decades, many new dental materials and ceramic systems have emerged. Yttrium-stabilized tetragonal zirconia polycrystalline (Y-TZP) ceramics are now favored in prosthetic dentistry for their excellent mechanical properties, biocompatibility, and aesthetic qualities. Zirconia restorations are mainly of two types: those with feldspathic porcelain and those made entirely of monolithic zirconia. Monolithic zirconia, known for its high translucency and strong mechanical properties, often uses yttria (Y₂O₃) as a stabilizer [8].

Zirconia is manufactured using two computer-aided design/computer-aided manufacturing (CAD/CAM) techniques: soft machining and hard machining. In soft machining, pre-sintered zirconia blocks are shaped and then sintered to achieve maximum strength, resulting in 25% shrinkage. In hard machining, fully sintered zirconia blanks are milled to the desired dimensions [9]. The color can be adjusted by adding metal oxides to the zirconia powder or immersing the framework in metal salt solutions. Monolithic restorations, created using CAD/CAM technology, can be finished by polishing or applying a glaze layer. Even with accurate manufacturing, occlusal adjustments are often needed when placing prostheses, and the occlusal surface must be properly polished afterward [10]. A rough zirconia surface can lead to increased enamel wear, and moisture exposure may cause low-temperature degradation (LTD) of the zirconia. A well-polished zirconia surface is less abrasive to opposing teeth, has better biocompatibility, and reduces bacteria and plaque buildup. Due to zirconia’s uniform polycrystalline structure and high hardness, regular polishing methods are ineffective. As a result, specialized polishing systems using high-hardness particles, such as diamond, have been developed [11].

Currently, no standardized method exists for finishing and polishing monolithic zirconia restorations, and the effects on their mechanical and optical properties are not well-documented. Therefore, this review aims to evaluate the impact of finishing and polishing on the mechanical strength and color properties of zirconia.

History of zirconia

In the 18th century, German chemist Martin Heinrich Klaproth identified zirconium by extracting zirconium oxide from the gemstone jacinth. Swedish chemist Jöns Jakob Berzelius subsequently isolated the metallic form of zirconium [12]. For the next 150 years, zirconia was mainly used for heavy-duty bricks and special high-refractive-index glass. The biomedical properties of zirconia were first studied by Helmer and Driskell in 1969. In 1972, Garvie and Nicholson discovered that adding oxides such as calcium, yttria, and magnesia to zirconia could stabilize its tetragonal phase, preventing it from changing to the monoclinic phase, and improving its resistance to cracking. As aesthetics became more important in dentistry, there was a search for materials that were both strong and durable, offering an alternative to metal. In the early 1960s, porcelain jacket crowns were made with feldspathic porcelain and reinforced with an alumina core. However, their low compressive strength limited their use to non-esthetic areas.

In 1963, the porcelain-fused-to-metal (PFM) crown was developed and became the standard for crowns and bridges for many years. A key issue with PFM crowns was the metal substructure, which required an opaque layer before adding the veneering ceramic. The opaque layer in PFM crowns blocks light, affecting the crown's hue and value, especially at the gingival margin, which can make the cervical third appear gray if there is no porcelain margin. In contrast, all-ceramic crowns transmit light, maintaining the natural tooth color even in thinner sections, which enhances their aesthetic appeal. In the 1980s, glass ceramics like Dicor were introduced. Dicor, made using the lost wax technique, was visually appealing but fragile and primarily used for anterior teeth. The 1990s brought advancements with the CEREC milling machine, which could create ceramic inlays from solid blocks. During this period, new alternatives including Empress ceramics, In-Ceram, and Procera were also introduced. Procera used high pressure and temperature to sinter a pressed aluminum core, producing a dense, strong alumina oxide core that could be veneered with porcelain. Before this, crowns made from sintered alumina oxide were the strongest non-metal option available. In the 2000s, the introduction of CAD/CAM systems revolutionized ceramic restorations by incorporating advanced, toughened zirconia [13-15].

## Review

Monolithic zirconia

PFM crowns often cause a grayish appearance at the cervical margin due to light reflecting off the metal. This led to a shift toward all-ceramic materials like zirconia, which are veneered with translucent feldspathic or glass-ceramic materials for better aesthetics. However, zirconia layered crowns are prone to porcelain chipping at the core-veneer interface. Ceramic chipping can be avoided by using full-contour or monolithic zirconia crowns, which have excellent mechanical properties and avoid cohesive veneering failure. To counteract zirconia's natural opacity, it can be colored before sintering for a more natural look. Monolithic zirconia offers high flexural strength, requires less invasive dental preparation, reduces wear on opposing teeth, has good aesthetics, and avoids chipping [16]. Although earlier monolithic zirconia had low aesthetic performance due to poor transparency, recent advancements have improved translucency while maintaining strength. Monolithic crowns are recommended for patients with challenging occlusion, damaged occlusal surfaces, parafunctional habits, a history of fractures, or limited space for restorative materials. They provide good aesthetics for molars but are less suitable for anterior teeth where a natural appearance is essential, as they can appear opaque compared to layered feldspathic restorations. To enhance their appearance, monolithic zirconia can be colored by mixing metallic pigments into the zirconia powder or by dipping the crowns in solutions containing rare earth elements. Recent studies on monolithic zirconia have examined various factors, including surface treatments, wear on the material and antagonists, fracture resistance, surface roughness, compressive and flexural strength, chipping resistance, hardness, elastic modulus, and color stability. While in vitro results may not fully capture clinical performance yet, they have provided valuable insights into monolithic zirconia's performance (Table 1) [17].

**Table 1:** Comprehensive classification of dental ceramic based on composition and structure PFM: porcelain-fused-to-metal; YSZ: yttrium-stabilized zirconia; PSZ: partially stabilized zirconia Credit: Maaz Vohra

Category	Subcategory	Composition	Properties
By composition			
Glass-based ceramics	Feldspathic porcelain	Silica (SiO₂), alumina (Al₂O₃), potassium oxide (K₂O), and soda (Na₂O)	High esthetic value and low mechanical strength
	Leucite-reinforced ceramics	Glass matrix with leucite crystals (KAlSi₂O₆)	Enhanced strength and moderate esthetics
	Lithium disilicate	Lithium disilicate crystals (Li₂Si₂O₅) within a glass matrix	High strength and high translucency
	Glass-infiltrated ceramics	Porous ceramic core infiltrated with glass	Increased strength and moderate translucency
Non-glass-based ceramics	Alumina-based ceramics	Alumina (Al₂O₃)	High mechanical strength and moderate translucency
	YSZ	Zirconia (ZrO₂) stabilized with yttrium oxide (Y₂O₃)	High mechanical strength and good toughness
	PSZ	Zirconia partially stabilized with other oxides	Enhanced toughness and variable translucency
	Fully stabilized zirconia	Fully stabilized zirconia with high density	Superior strength and durability
				By structure			
				Monolithic ceramics	Monolithic zirconia	Single material without layering	High mechanical strength and low translucency
Monolithic lithium disilicate	Single material optimized for esthetics	High strength and high translucency		
Layered ceramics	PFM	Metal substructure covered with porcelain	Strong but metal substructure affects esthetics
	Porcelain-layered zirconia	Zirconia core covered with a porcelain layer	Combines high strength with improved esthetics
	Porcelain-layered alumina	Alumina core covered with a porcelain layer	High strength and improved esthetics

Finishing and polishing monolithic zirconia

A crucial factor in selecting dental crown materials is their potential to wear down enamel. The restorative material should mimic the microstructural and bio-tribological properties of natural teeth to prevent excessive enamel abrasion. Full-contour zirconia crowns are treated with various methods, including grinding, polishing, glazing, and heating, to protect opposing teeth from damage. Research has found that polishing feldspathic porcelain can serve as an alternative to glazing and that surface treatments can influence the color and aesthetics of porcelain restorations by altering grain size and pore distribution. Moreover, monolithic zirconia crowns are subjected to thermal fluctuations in a moist environment during their use, which can affect their structure, leading to changes in color and reduced mechanical properties [18].

Polishing

Sequential polishing of zirconia involves using various diamond points, rubber wheels, and abrasive pastes to achieve a smooth and lustrous surface without adding any layers, unlike glazing. The process consists of three stages: coarse finishing, intermediate polishing, and final polishing. Coarse finishing uses rough-grit tools to shape the restoration and remove excess material. Intermediate polishing employs medium-grit tools to smooth out grooves and scratches left from the coarse finishing. Final polishing uses fine-grit tools to create a smooth, enamel-like finish with high light reflectivity. To achieve the best results, it is essential to use the tools in sequence, starting with coarse and progressing to finer grits, while varying the rotations per minute [19].

Glazing

**Figure 1:**
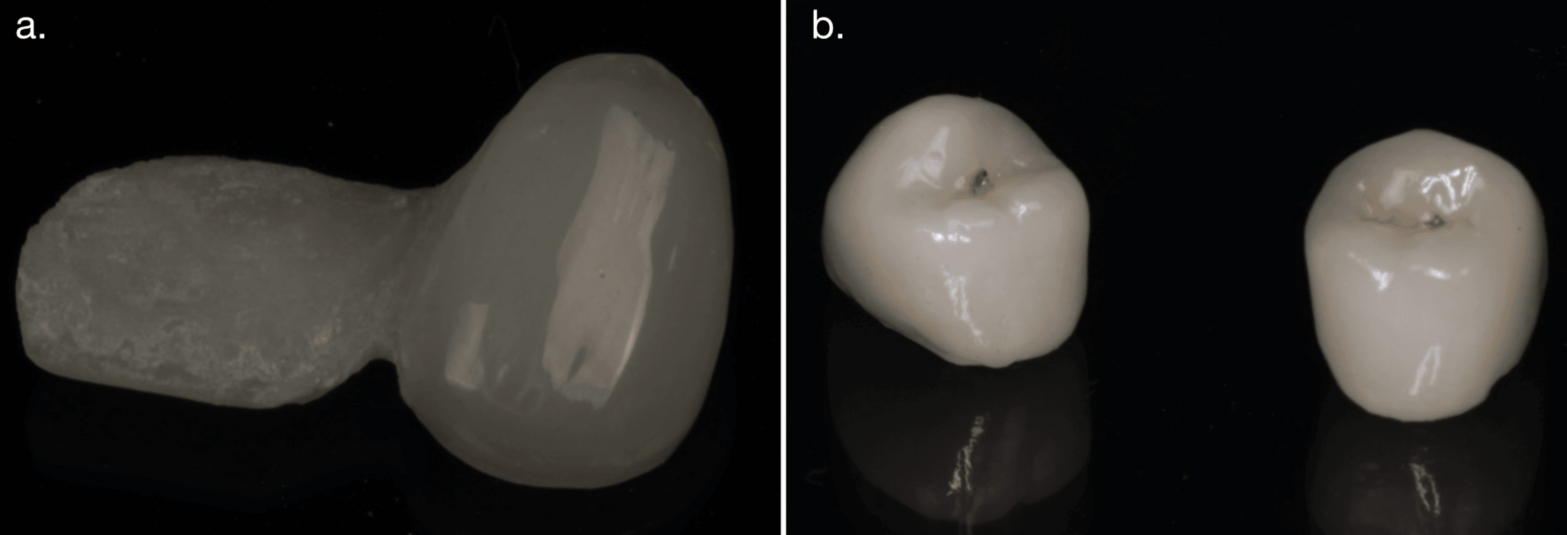
a) Sequentially polished monolithic zirconia and b) glazed zirconia Image credit: Maaz Vohra

Glazing can be done by applying a thin coat of transparent glass to the surface and firing it or by heating the restoration to a temperature that produces a shiny finish. While glazing is commonly used to restore a high-gloss surface, it is reported that these glazed layers can wear off within six months (Figure 1) [20].

Effect of finishing and polishing on clinical properties of monolithic zirconia

**Figure 2:**
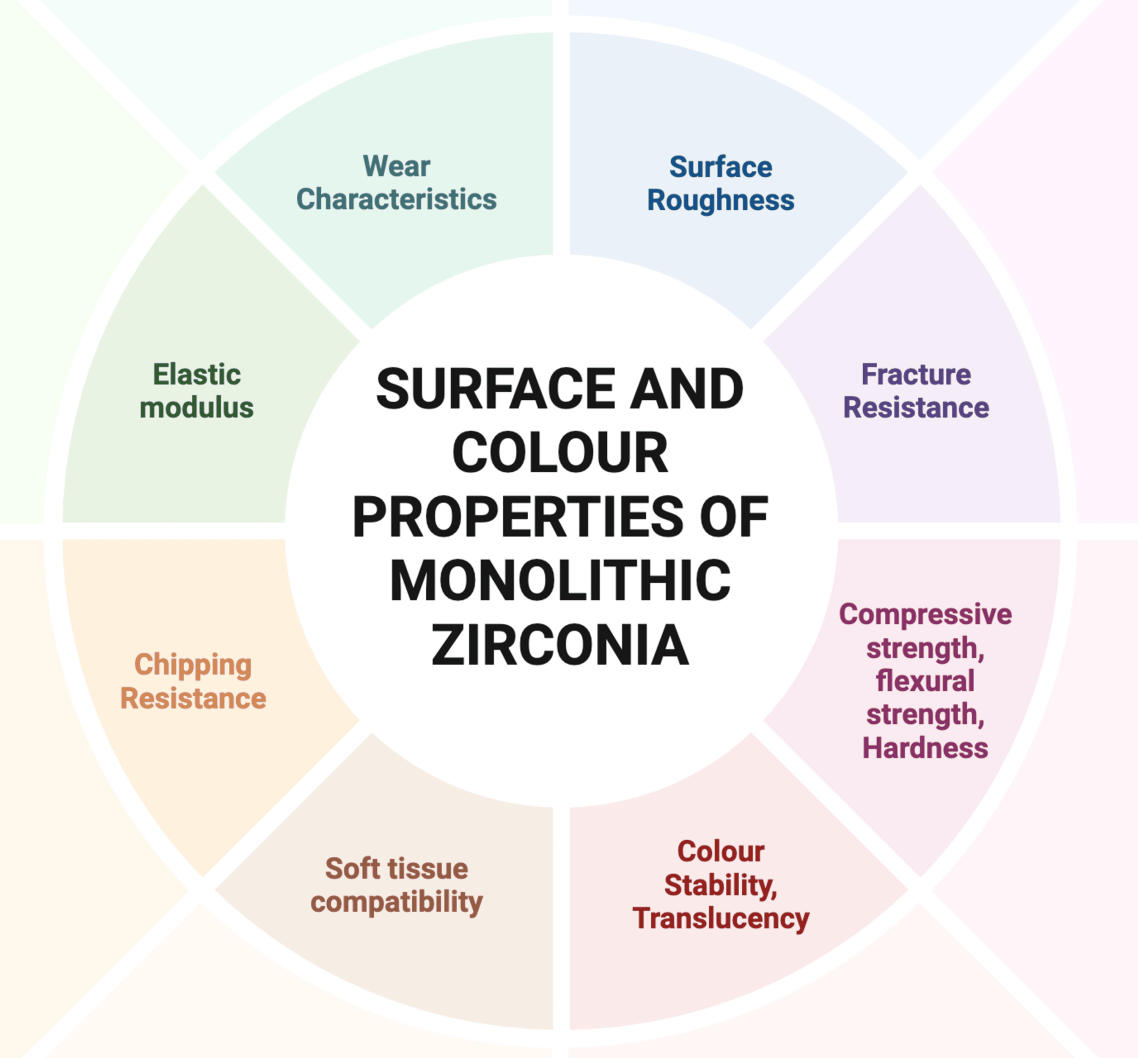
Surface and color properties of monolithic zirconia Image credit: Amrutha Shenoy

The finishing and polishing of monolithic zirconia affect its clinical properties, including wear characteristics, surface roughness, fracture resistance, strength, hardness, color stability, translucency, and soft tissue compatibility (Figure 2).

Wear characteristics

Research has explored the impact of various surface treatments of monolithic zirconia on the wear of opposing teeth or restorations. The findings revealed that polished zirconia caused less wear on hydroxyapatite than glazed zirconia. Another study using an Alabama wear-testing device discovered that polished zirconia caused minimal wear on opposing enamel. In contrast, glazed, polished, and reglazed zirconia resulted in more wear, with the most significant enamel wear occurring from veneering porcelain [21]. Overall, polished zirconia was found to be more wear-friendly for opposing teeth. Additionally, tests showed that polished zirconia caused the least wear on steatite balls, while glazed zirconia caused the most. Further research compared the wear on enamel and feldspathic porcelain after chewing against heat-pressed ceramics and feldspathic porcelain. The study found that monolithic zirconia caused much less enamel wear compared to glass-ceramic. Polished zirconia also resulted in less enamel wear than glazed zirconia when tested using a chewing simulator. The conclusion was that polished zirconia is more effective at reducing wear on opposing teeth [22]. In both three-body and two-body wear tests, polished zirconia caused less wear on opposing surfaces compared to glazed zirconia. Polished zirconia, whether treated manually or mechanically, results in less enamel wear compared to glazed zirconia, but can cause more enamel cracks. Glazed zirconia caused more wear on Empress crowns than machined zirconia, but its effects were similar to those of e-max. In contrast, a study by Amer et al. found that polished zirconia resulted in more wear on stainless steel antagonists than glazed or veneered zirconia. Overall, surface treatment significantly affects both antagonist wear and the restorative material's durability [23].

Surface roughness

Surface roughness significantly impacts wear on the antagonist’s tooth and is primarily influenced by the surface treatment of zirconia. Studies have shown that glazed zirconia is smoother than zirconia finished with a diamond bur or polished zirconia. While glazed zirconia and zirconia that has been polished and then glazed are smoother than enamel and veneered zirconia, they are rougher than zirconia polished alone. Among different brands of polished zirconia, monolithic zirconia exhibits higher roughness than e-max but lower roughness than feldspathic porcelain. Untreated machined zirconia is rougher than glazed zirconia. Although the difference in roughness between glazed zirconia and highly polished monolithic zirconia is not statistically significant, polished zirconia generally has a smoother surface. Grinding zirconia reduces surface roughness, whereas polishing increases it. Therefore, polishing zirconia after intraoral adjustments is crucial to minimize wear on opposing teeth [24].

Fracture resistance

Studies show that monolithic zirconia is very durable, even when made thin, making it suitable for high-stress areas. Research on three-unit zirconia FPDs, with or without porcelain, found similar fracture strengths between 1173.5 and 1316.0 N. This demonstrates that monolithic zirconia is a strong alternative to veneered zirconia. Monolithic zirconia had the highest fracture resistance (5.62 N) under axial loading compared to monolithic lithium disilicate and feldspar ceramics. Polished, sintered, and glazed zirconia all supported more weight than veneered zirconia, with polished and glazed zirconia performing similarly. However, sintered zirconia had a lower load-bearing capacity compared to polished and glazed zirconia, both of which could handle fracture loads over 10,000 N. The thickness of the zirconia also affects its strength: 1.5 mm thick monolithic zirconia withstood a fracture load of 4109.93±610.18 N, and this load decreased with thinner samples. Monolithic zirconia with a 0.5 mm occlusal thickness had a higher fracture load (5558±522 N) than lithium disilicate crowns with a 1.5 mm thickness (3147±409 N) [25]. The fracture resistance of monolithic zirconia is unaffected by axial thickness, but autoclaving reduces its fracture load from 5683 to 3975 N, with minimal impact from cyclic loading. Implant-supported monolithic zirconia crowns have a higher fracture resistance (6065 N) compared to veneered lithium disilicate crowns (2788 N). Monolithic zirconia brands vary in fracture resistance from 2795 to 3038 N, while heat-pressed lithium disilicate, porcelain-veneered zirconia, and high-translucency zirconia have lower resistances of 1856 N, 2229 N, 1480 N, and 1808 N, respectively [26,27]. The literature shows that monolithic zirconia remains highly fracture-resistant even at thin layers, making it ideal for areas that endure heavy stress. In contrast, veneered zirconia is prone to chipping, which limits its suitability for such applications.

Compressive strength, flexural strength, chipping resistance and elastic modulus

Study found that monolithic zirconia had better chipping resistance and flexural strength than glass-infiltrated zirconia, lithium disilicate, and veneering porcelain. Another study showed that full-contour zirconia crowns had higher flexural strength, compressive strength, and elastic modulus than composite resins and lithium disilicate. Research found that sintering temperature did not alter the flexural strength of monolithic zirconia, indicating it is a promising choice for durable and attractive restorations [28].

Hardness

A study measuring the mean hardness of nine CAD/CAM materials found that monolithic zirconia had the highest hardness. Although higher hardness might suggest a greater potential for wear on opposing teeth, it is not the only factor. The wear of antagonist teeth is also influenced by surface roughness in addition to hardness [29].

Color stability

Discoloration of intrinsic materials can result from both internal and external factors. Internally, changes in the resin matrix or its interaction with fillers often cause discoloration. Externally, colorants from food, drinks, or smoking can adsorb or absorb into the material, with the extent of discoloration influenced by oral hygiene and personal habits. This discoloration can be assessed visually or using instrumental methods. Color measurement systems like Munsell and CIE-Lab quantify color based on L* (brightness), a* (red, green), and b* (yellow, blue). Thermal stress from temperature changes in the mouth can lead to mechanical damage in zirconia ceramics. A study found that increasing the amount of coloring liquid on monolithic zirconia reduced brightness and opalescence while making it more yellow. The largest color change (ΔE*ab of 15.7) was observed between untreated zirconia and zirconia with five applications of coloring liquid. Another study using reflection spectrophotometry revealed that both polishing and glazing reduced zirconia's lightness, with glazing causing it to become more yellowish compared to polishing. In conclusion, surface treatments and coloring applications significantly alter the color properties of full-contour zirconia restorations, so clinicians should carefully consider these factors when selecting the shade for monolithic zirconia [30,31].

Translucency

Studies have examined light transmission through zirconia with various surface treatments and conditions. Research showed that polished monolithic zirconia allows more light to pass through compared to glazed or layered zirconia. Additionally, monolithic zirconia transmits less light than lithium disilicate. Another study found that increasing both the sintering time and temperature enhances the translucency of monolithic zirconia [32].

Soft tissue compatibility

Cell and tissue adhesion is crucial for developing effective biomaterials, particularly in dentistry. Cell adhesion on ceramics depends on surface roughness, wettability, and chemical composition. Studies found partially stabilized zirconia had better fibroblast adhesion than monolithic zirconia. Zirconia also reduced bacterial growth more effectively than titanium surfaces [33]. The properties reviewed and the respective included literature have been summarized in Table 2.

**Table 2:** Included studies assessing various parameters associated with monolithic zirconia after polishing and glazing YSZ: yttrium-stabilized zirconia

Authors	Study design	Objective	Conclusion
Gergo Mitov et al. (2018) [1]	In vitro study	To assess how various finishing methods impact the wear of zirconia compared to natural enamel	Polished zirconia caused the least enamel wear, similar to the glass-ceramic control. Surface roughness before testing did not significantly affect the amount of enamel wear
Zhang Y et al. (2013) [16]	In vitro study	To evaluate how different surface treatments, grinding, polishing, and glazing, affect the occlusal surface of fully-stabilized zirconia restorations and their fatigue performance	Polishing and glazing effectively smoothed the surface and reduced defects and roughness similarly. These treatments did not affect fatigue failure load, cycles to failure, or survival rates. Both are recommended for reducing surface roughness without impacting fatigue performance
Aljomard YR et al. (2022) [34]	Systematic review and meta analysis	To assess the impact of monolithic zirconia restorations on dental enamel wear	The findings indicated that enamel wear caused by monolithic zirconia fell within acceptable statistical limits. Additionally, polished monolithic zirconia led to less enamel wear compared to glazed zirconia surfaces
Ghaffari T et al. (2022) [35]	In vitro study	To evaluate the three-body wear of enamel against three ceramics, dense sintered YSZ, lithium disilicate, and conventional low-fusing feldspathic porcelain, each treated with rough, smooth, or glazed surfaces.	The data indicated that dense sintered YSZ resulted in the least enamel wear, whereas glazed zirconia caused the most wear on opposing enamel
Kim HK et al. (2013) [36]	In vitro study	To evaluate how polishing and glazing impact the color and spectral properties of monolithic zirconia	Polishing monolithic zirconia results in a noticeable color change, reducing its lightness. Similarly, glazing also reduces lightness but adds a yellowish hue to the zirconia
Kim HK et al. (2016) [37]	In vitro study	To examine how different surface treatments affect the translucency, opalescence, and surface texture of dental monolithic zirconia ceramics	Surface treatments showed no major differences in translucency. Glazing, however, significantly reduced opalescence and surface roughness. SEM images indicated that surface treatments altered the texture of monolithic zirconia ceramics
da Rosa LS et al. (2023) [38]	Systematic review and meta analysis	To assess how post-processing methods impact the surface roughness and flexural strength of YSZ ceramics following grinding	Polishing is the most effective post-processing treatment for YSZ ceramics after grinding, as it enhances flexural strength and smooths the surface.
Cochis A et al. (2020) [39]	In vitro and In vivo study	To assess the colonization of microbes on new ceramic materials	In vitro, zirconia attracted more *S. mutans* than titanium disks, while titanium attracted more *S. sanguis*. In vivo, zirconia accumulated fewer bacteria overall and fewer potential pathogens compared to titanium

## Conclusions

This review examines how polishing and finishing methods affect the appearance and performance of monolithic zirconia restorations. Polishing and glazing effectively smooth zirconia surfaces, with polishing proving to be superior in reducing surface roughness and minimizing wear on opposing teeth. Although both treatments improve surface quality, they do not significantly differ in their impact on fatigue resistance or load-bearing capacity. Polished monolithic zirconia exhibits high fracture resistance, flexural strength, and chipping resistance, making it suitable for load-bearing applications. While both polishing and glazing reduce lightness, polishing maintains better translucency compared to glazing. Overall, surface treatments enhance the aesthetic and clinical performance of monolithic zirconia, supporting its use in restorative dentistry for achieving optimal functional and visual results.
